# Characteristics of patients showing dislocation after total hip arthroplasty in an acute care hospital: A retrospective cohort study

**DOI:** 10.1097/MD.0000000000042664

**Published:** 2025-06-06

**Authors:** Fumihiko Suenaga, Tokio Kinoshita, Yoshinori Yasuoka, Kohei Minami, Yukihide Nishimura, Daisuke Nishiyama, Hiroshi Yamada, Ken Kouda

**Affiliations:** a Department of Rehabilitation Medicine, Wakayama Medical University, Wakayama, Japan; b Department of Rehabilitation Medicine, Chuzan Hospital, Okinawa, Japan; c Division of Rehabilitation, Wakayama Medical University Hospital, Wakayama, Japan; d Department of Rehabilitation Medicine, Iwate Medical University, Iwate, Japan; e Department of Orthopedic Surgery, Wakayama Medical University, Wakayama, Japan.

**Keywords:** acute phase, complications, dislocation prevention, risk factor, total hip arthroplasty

## Abstract

Most previous studies on dislocation after total hip arthroplasty (THA) have focused on patient- and surgery-related factors without detailing the specific circumstances of dislocation events. Therefore, this study aimed to analyze dislocation cases and compare these relevant factors between patients who experienced dislocation and those who did not to inform targeted prevention measures during acute care hospitalization. This retrospective study examined the electronic medical records of 420 patients (445 joints) who underwent THA and rehabilitation at our hospital between April 1, 2018, and August 31, 2023. A total of 9 cases of dislocation in 6 patients were reported. Dislocation occurred most frequently in bed within the patient’s room (5 cases; 55.6%), followed by in the rehabilitation room and the toilet (1 case each; 11.1%), and unknown locations (2 cases; 22.2%). Bed-related dislocations were associated with trunk rotation or external rotation of the hip joint; in 2 cases, the dislocation was already present when the patient awoke. The revision THA and THA indication rates, preoperative Functional Independence Measure scores, and locomotion status differed significantly between the patients who showed dislocation and those who did not. Revision THA, THA indications other than osteoarthritis, low Functional Independence Measure score at admission, and low locomotion status may increase the risk of dislocation. Furthermore, the findings suggest that dislocation prevention strategies during acute hospitalization should prioritize safe bed use and sleep positions.

## 1. Introduction

Dislocation is a serious adverse event following total hip arthroplasty (THA), and, along with mechanical loosening, is a leading cause for revision surgery.^[1]^ In addition to prolonging hospitalization in patients indicated for revision THA,^[2]^ additional treatment costs associated with dislocation have increased over the years and represent a considerable financial burden on healthcare systems. Specifically, the average costs for treatment in the United States were $54,553 in 2009 and $77,851.24 in 2017.^[1,3]^ Dislocations occur frequently within weeks after surgery and 60% of dislocations occur within the first 5 weeks,^[4]^ with the median time to dislocation being 24 (12–45) days^[5]^ and the time to readmission being 40.0 (15.3–92.8) days.^[6]^ Therefore, given their frequency and impact, early dislocation prevention is essential not only to reduce patient morbidity but also to mitigate healthcare costs.

Previous studies on post-THA dislocation have focused on patient-related factors, such as age, sex, body mass index (BMI), THA indications, and history of spine fusion surgery, as well as surgery-related factors, including the approach method and cup-placement angle.^[7–19]^ These investigations have made it possible to identify patients at high risk of dislocation. However, to develop effective dislocation prevention protocols and optimize the inpatient environment post-THA, it is necessary to clarify the specific circumstances under which dislocations occur. Yet to date, the locations and circumstances of dislocations occurring during hospitalization shortly after THA have not been investigated.

This study examined the characteristics of patients who experienced dislocation at our hospital and compared them between patients who experienced dislocation and those who did not to inform targeted prevention measures during acute care hospitalization.

## 2. Materials and methods

### 2.1. Study design and setting

This retrospective cohort study was conducted at Wakayama Medical University Hospital.

### 2.2. Participants

This study analyzed the electronic medical records of all patients who underwent THA and rehabilitation at our hospital between April 1, 2018, and August 31, 2023. Regarding the dislocation cases, reports submitted to the Medical Safety Promotion Department at our hospital were retrieved, and detailed information regarding each case was collected. The study period was defined as the acute period from the time of THA to the time of transfer from our hospital. We excluded patients who received a dual-mobility cup and those who underwent revision arthroplasty on only 1 side of the cup or the head.

### 2.3. Outcome measures

The survey items included the location of dislocation, dislocation status, direction of the dislocation, interval between surgery and dislocation, and treatment after dislocation. In addition, the Functional Independence Measure (FIM) score at admission,^[20]^ preadmission locomotion status, preadmission Devane Activity score,^[21]^ American Society of Anesthesiologists physical status classification system (ASA-PS) score at admission,^[22]^ Charlson Comorbidity Index (CCI) at admission,^[23]^ approach method, cup inclination angle/anteversion angle,^[24]^ cup size, head size, use of stem cement, history of spinal fusion surgery, number of days from surgery to discharge, and discharge destination were measured. The FIM consists of 18 items with a motor subscale (13 items) and a cognition subscale (5 items), each of which was assessed using a 7-point ordinal scale.^[20]^ The Devane Activity Score uses a 5-point scale that indicates a patient’s activity level (1 indicates a sedentary/dependent status, whereas 5 indicates participation in contact sports).^[21]^ The ASA-PS classifies the health status of patients before surgery into 6 classes (class I indicates a healthy person, whereas class VI indicates a brain-dead organ transplant donor).^[22]^ The CCI evaluates comorbidities that contribute to death, with higher CCI values indicating a higher predicted mortality rate.^[23]^

### 2.4. Data analyses

Values of the variables are presented as number, mean ± standard deviation, and median (25th–75th percentiles), where applicable. Comparisons of age, weight, height, BMI, FIM score, CCI, days from surgery to discharge, cup and head size, cup inclination angle, and anteversion angle in patients who experienced dislocation and those who did not were performed using the Mann–Whitney *U* test. The chi-square test was used to evaluate the percentage of primary versus revision surgeries, THA indication, ASA-PS score, Devane Activity score, discharge destination, presence of spinal fusion, approach method, and use of stem cement. Differences were considered statistically significant at *P* < .05, and statistical analyses were performed using GraphPad Prism 6 software (GraphPad Software Inc., San Diego, CA).

### 2.5. Ethical considerations

This study was conducted in accordance with the Declaration of Helsinki, and the protocol was approved by the relevant ethics review committee (number: 4100). This retrospective study posed no additional risks to patients during data collection and analysis, and all patient information was protected. Information concerning this study was posted on the university website, and patients or their families or relatives were given the opportunity to opt out of the study. The ethics review committee waived the requirement for written informed patient consent owing to the retrospective design of the study.

## 3. Results

**Figure 1.**
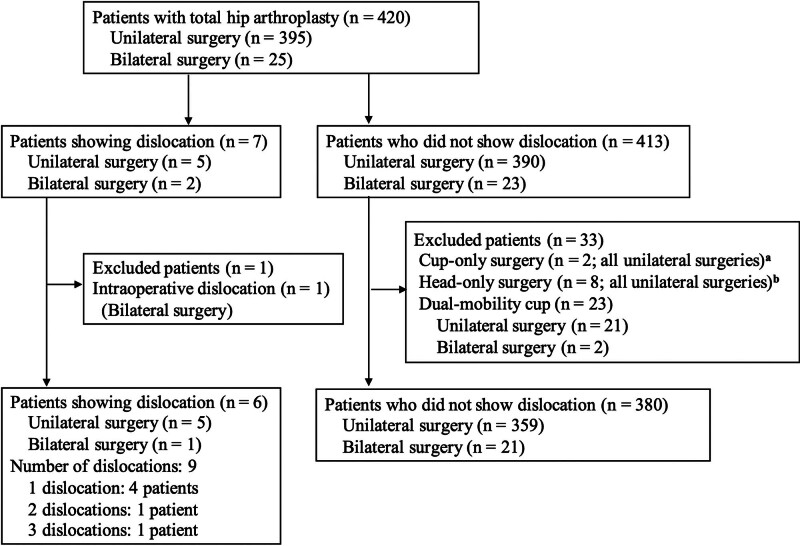
Patient flow diagram. (A, B) Unilateral revision was performed in all patients who underwent cup or head-only surgery.

Fig. 1 shows the flow diagram of patient selection. During the study period, THA was performed for 445 joints in 420 patients; among these, 7 patients experienced dislocation during hospitalization. After excluding 1 patient who experienced intraoperative dislocation, the dislocation group consisted of 6 patients. Four patients experienced dislocation once, one experienced it twice, and one experienced dislocation 3 times, yielding a total of 9 dislocations during hospitalization. The number of patients who did not experience dislocation was 413. After excluding 10 patients who underwent cup-only or head-only revision and 23 patients who received dual-mobility cups, the non-dislocation group consisted of 380 patients (401 joints).

The incidences of dislocation during the study period were 1.55% in patients who underwent THA (6/386 patients) and 1.47% in the joints for which THA was performed (6/407 joints). The incidences of dislocation were 0.79% (3/381 joints) after primary THA and 11.5% (3/26 joints) after revision THA.

Table 1 presents the characteristics of patients in the dislocation and non-dislocation groups. Age, sex, height, weight, and BMI were not significantly different between the 2 groups. The rate of revision THA in the dislocation group (3 patients, 50%) was significantly higher than that in the non-dislocation group (23 patients, 5.7%). The most frequent indications for THA were osteoarthritis (2 patients, 33.3%) and fracture (2 patients, 33.3%; both fractures around THA) in the dislocation group, and osteoarthritis (300 patients, 74.8%) and osteonecrosis (53 patients, 13.2%) in the non-dislocation group, with the 2 groups showing significant differences in the proportions of patients with these THA indications. In the dislocation group, the total FIM score before surgery was 85.9 (49.8–115.0), and the scores for the motor and cognition subscales were 55.5 (30.5–81.3) and 32.5 (18.8–35.0), respectively; the corresponding values in the non-dislocation group were 124 (117.0–125.3), 89.0 (82.0–91.0), and 35 (35.0–35.0), respectively. The dislocation group showed significantly lower scores for all measured items than the non-dislocation group. In the dislocation group, 3 patients (50%) walked with a cane and 2 (33.3%) required a wheelchair before hospitalization, whereas in the non-dislocation group, 168 (44.2) patients could walk without assistive devices and 168 (44.2%) walked with a cane before hospitalization, with the percentages differing significantly between the 2 groups. The groups showed no significant differences in the Devane Activity score, ASA-PS, or CCI. However, the interval from surgery to discharge was significantly longer in the dislocation group than in the non-dislocation group (37.8 ± 16.2 days vs 18.1 ± 6.5 days). The discharge destination also differed significantly between the groups, with 83.3% of the patients who experienced dislocation being transferred to other facilities and 65.3% of those without dislocations being discharged home. However, the 2 groups showed no significant difference in the proportions of patients who showed spinal fusion and those who did not.

**Table 1:** Patient characteristics.

	Patients with dislocation (n = 6)	Patients without dislocation (n = 380)	*P* value
Age (yr)	66.3 ± 12.4	68.2 ± 11.6	.6095
Male/female	2/4	72/308	.3744
Height (cm)	150.4 ± 10.6	153.9 ± 8.6	.3450
Weight (kg)	49.4 ± 10.7	56.4 ± 11.9	.1445
BMI (kg/m^2^)	22.0 ± 4.5	23.7 ± 4.0	.4970
Primary/revision*	3/3 (50.0%/50.0%)	378/23 (94.3%/5.7%)	<.0001
THA indication*			<.0001
Osteoarthritis	2 (33.3%)	300 (74.8%)	
Fracture	2 (33.3%)	9 (2.2%)	
Osteonecrosis	1 (16.7%)	53 (13.2%)	
Post-THA infection	1 (16.7%)	4 (1%)	
THA loosening	0	16 (4.0%)	
Developmental dysplasia	0	9 (2.2%)	
Rheumatoid arthritis	0	7 (1.7%)	
Tumor	0	2 (0.5%)	
THA dislocation	0	1 (0.25%)	
Total FIM score	85.9 (49.8–115.0)	124.0 (117.0–125.3)	
Motor subscale score	55.5 (30.5–81.3)	89.0 (82.0–91.0)	
Cognition subscale score	32.5 (18.8–35.0)	35.0 (35.0–35.0)	
Locomotion status			.0042
Possible without aids	0	168 (44.2%)	
With a cane	3 (50.0%)	168 (44.2%)	
With walking frame	1 (16.7%)	6 (1.6)	
Using wheelchair	2 (33.3%)	38 (10.0%)	
Devane Activity Score			.2223
1	3 (50%)	69 (18.2%)	
2	3 (50%)	159 (41.8%)	
3	0	71 (18.7%)	
4	0	76 (20.0%)	
5	0	5 (1.3%)	
ASA-PS			.9286
Ⅰ	3 (50.0%)	194 (51.1%)	
Ⅱ	3 (50.0%)	162 (42.6%)	
Ⅲ	0	22 (5.8%)	
Ⅳ	0	2 (0.5%)	
Ⅴ	0	0	
Ⅵ	0	0	
CCI	0.5 (0.0–1.8)	0.0 (0.0–1.0)	.4764
Days from surgery to discharge	37.8 ± 16.2	18.1 ± 6.5	<.0006
Discharge destination			
Home/other facilities	1/5 (16.7%/83.3%)	248/132 (65.3%/34.7%)	.0136
Spine fusion	1 (16.7%)	17 (4.5%)	.1599

The FIM score and CCI are shown as median (25th–75th percentile).

BMI = body mass index, THA = total hip arthroplasty, FIM = Functional Independence Measure, ASA-PS = American Society of Anesthesiologists physical status, CCI = Charlson Comorbidity Index.

* The results for the dislocation group were based on the data obtained for 6 dislocated joints, and those for the non-dislocation group were based on the data for 401 joints that were treated with THA. The Mann–Whitney *U* test was used to compare age, height, weight, BMI, FIM score, CCI, and days from surgery to discharge between patients who experienced dislocation and those who did not, and the chi-square test was used to evaluate the percentage of primary versus revision surgeries, locomotion status, Devane score, ASA-PS score, discharge destination, and the presence of spinal fixation.

Table 2 summarizes the dislocation scenarios. Six patients experienced 9 dislocations. The dislocations occurred on the bed in the patient’s own room in 5 cases (55.6%), the rehabilitation room and the toilet in 1 case each (11.1%), and at an unknown location in 2 cases (22.2%), with dislocation on the bed accounting for half of the cases. The dislocation direction was anterior in 6 cases (66.6%) and posterior in 3 cases (33.3%). The interval from surgery to dislocation ranged from 4 to 22 days (mean, 11.9 ± 5.7 days). Dislocation occurred during range of motion training in the hip extension direction by a physical therapist in 1 case; when standing up from a toilet seat with the hip in external rotation in 1 case; when the patient moved in an escape reaction on experiencing pain as a result of the balloon catheter getting stuck while the patient was on the bed in 1 case; when the patient tried to pick up an object from a sitting posture on the bed and rotated the trunk in 1 case; when the patient externally rotated the hip joint while changing a lower garment during 1 case; and in unknown circumstances in 4 cases, including 2 cases in which dislocation was identified on routine radiographic assessments and 2 cases in which dislocation was observed upon waking. Treatment after dislocation was closed reduction in 2 of the 4 patients who experienced a first dislocation, revision THA in the other 2 patients, and revision THA after the last dislocation in 2 patients who experienced multiple dislocations.

**Table 2:** Summary of the cases showing dislocation.

	THA indication	Approach	Primary/revision	Location of dislocation	Direction of dislocation	Interval from surgery to dislocation	Dislocated situation	Treatment
Patient A								
1^st^	Osteoarthritis	T	Primary	Rehabilitation room	Anterior	12	During right hip extension range of motion training by therapist	Closed reduction
2^nd^				Unknown	Anterior	19	Unknown (periodic radiographic evaluation revealed dislocation)	Revision THA
Patient B								
1^st^	Fracture	P	Revision	Unknown	Posterior	7	Unknown (periodic radiographic evaluation revealed dislocation)	Revision THA
Patient C								
1^st^	Osteoarthritis	T	Primary	Toilet	Anterior	10	When standing up from a toilet seat with the hip in external rotation	Revision THA
Patient D								
1^st^	Infection	P	Revision	On the bed	Posterior	22	Unknown (dislocated upon awakening)	Closed reduction
Patient E								
1^st^	Fracture	L	Revision	On the bed	Anterior	9	During pain escape movement	Closed reduction
2^nd^				On the bed	Anterior	10	Unknown (dislocated upon awakening)	Closed reduction
3^rd^				On the bed	Posterior	14	When the patient rotated the trunk to pick up an object in a sitting position	Revision THA
Patient F								
1^st^	Necrosis	ALS	Primary	On the bed	Anterior	4	When changing lower garments on the bed	Closed reduction

ALS = anterolateral supine, L = lateral, P = posterior, T = two-incision, THA = total hip arthroplasty.

Table 3 shows the surgical information for both groups. The surgical approaches employed were the anterior lateral supine + Orthopadisehe Chirurgie Munchen approach in 287 cases, 2-incision approach in 98 cases, posterior and lateral approaches in 9 cases each, and anterior approach in 4 cases. The surgical approaches in the cases of dislocation were the posterior (22.2%), lateral (11.1%), 2-incision (2.0%), and anterior lateral supine + Orthopadisehe Chirurgie Munchen (0.3%) approaches, with the incidence of dislocation differing significantly depending on the approach. The cup sizes were 51.3 ± 4.1 mm in the dislocation group and 48.8 ± 3.3 mm in the non-dislocation group, and the femoral head sizes were 32.7 ± 3.0 mm in the dislocation group and 32.5 ± 2.0 mm in the non-dislocation group, with both sizes showing no significant intergroup differences. The dislocation rates of the stem were 0.85% when cement was not used and 5.8% when cement was used; the dislocation rate differed significantly in relation to cement usage. Cup inclination values were 47.3 ± 6.0° in the dislocated group and 45.2 ± 28.8° in the non-dislocated group, and cup anteversion values were 15.5 ± 4.7° in the dislocated group and 17.9 ± 6.9° in the non-dislocated group, with no significant differences between the 2 groups.

**Table 3:** Surgical information.

	Total (n = 407)	Cases showing dislocation (n = 6)	Cases without dislocation (n = 401)	Rate of dislocation (%)	*P* value
Approach					<.0001
ALS + OCM	287 (70.5%)	1	286	0.3	
Two-incision	98 (24.1%)	2	96	2	
Posterior	9 (2.2%)	2	7	22.2	
Lateral	9 (2.2%)	1	8	11.1	
Anterior	4 (1.0%)	0	4	0	
Cup size (mm)	48.8 ± 3.3	51.3 ± 4.1	48.8 ± 3.3		.1009
Femoral head size (mm)	32.5 ± 2.0	32.7 ± 3.0	32.5 ± 2.0		.7033
Femoral stem					.0059
Cementless	355 (87.2%)	3	352	0.85	
Cemented	52 (12.8%)	3	49	5.8	
Cup inclination (°)	45.2 ± 28.6	47.3 ± 6.0	45.2 ± 28.8		.1519
Cup anteversion (°)*	17.9 ± 6.8	15.5 ± 4.7	17.9 ± 6.9		.3519

Chi-square test was used to evaluate the approach method, and use of stem cement between patients who experienced dislocation and those who did not, and the Mann–Whitney *U* test was used to compare cup and head size, cup inclination angle, and anteversion angle.

ALS = anterolateral supine, OCM = Orthopadisehe Chirurgie Munchen, THA = total hip arthroplasty.

* Measurements were difficult to obtain in 20 joints, so the findings represent the data for 381 joints.

## 4. Discussion

The results of this study showed that more than half of the early postoperative THA dislocations occurred while the patient was on the bed, and that revision THA, THA indications, FIM score on admission, and preoperative locomotion status were related to the occurrence of dislocation. In addition, more than half of the cases of dislocation required revision THA, which increased the duration of hospitalization by a factor of 2 and decreased the percentage of patients who were discharged. To our knowledge, this study is the first to report specific dislocation conditions in addition to the factors related to the occurrence of dislocation in patients early after THA.

A meta-analysis spanning nearly 60 years and encompassing 5,030,293 patients with follow-up data exceeding 1 year reported that the incidence of dislocation after primary THA was 1.7%, with a 1-year dislocation rate of 1.0%.^[25]^ In contrast, the incidences of dislocation in cases of revision THA were reported to be 5.1% at 1 year postoperatively,^[26]^ 9.0% in broader reviews covering follow-ups from several months to years,^[27]^ and 15.9% in a 15-year follow-up study.^[28]^ The present results showed that the incidences of dislocation were 0.79% after primary THA and 11.5% after revision THA, but this study had a shorter study period than that of other previous studies and it evaluated the acute postoperative care period. Furthermore, the approach method, patient severity, indications, and other factors cannot be matched and compared to those used in previous studies; therefore, caution must be exercised when interpreting the results.

Anterior and 2-incision approaches are associated with a higher risk of dislocation in the direction of hip extension and external rotation, whereas lateral and posterior approaches show a higher risk of dislocation in the direction of flexion and internal rotation.^[29]^ Excluding 2 cases in which the occurrence status was unknown (patients A and B), most cases (71.4%) involved dislocations on the bed in the patient’s own hospital room. Surprisingly, 2 cases of dislocation occurred during sleep and did not involve large body movements. Although the circumstances of the dislocation are unknown, patient D showed posterior dislocation after undergoing a posterior approach, possibly because the hip joint involuntarily moved in the direction of flexion and internal rotation while sleeping. However, in patient E, anterior dislocation occurred despite a lateral approach. This patient had previously undergone rotational osteotomy, 2 rounds of plate fixation due to a fracture caused by a fall, 2 rounds of THA, and treatment for a fracture and wound infection before this surgery. Therefore, the anterior dislocation was thought to have occurred because the soft tissue was weak after surgery and treatment. Patient E also experienced 2 dislocations on bed, including a posterior dislocation. Therefore, in patients who have a history of multiple surgeries due to fractures, infections, or dislocations, care should be taken since the approach may not indicate the direction of dislocation. Patient F, who underwent an anterior approach, experienced dislocation during external rotation of the hip when putting on and taking off pants in bed, while patient C, who also underwent an anterior approach, experienced dislocation when standing up in the external rotation position of the hip during toileting. Patient C’s preoperative hip extension was −45°, and her range of motion at the time of dislocation was about the same, indicating that impingement may have occurred due to extension and external rotation of the hip joint when she stood up. These findings indicate that in the acute stage, patient education on bed mobility and proper sleep positioning is essential. Moreover, in patients who have a complex medical history and functional impairment, as in patients C and E, standardized dislocation precautions based solely on the surgical approach may be inadequate, underscoring the need for individualized guidance.

Revision THA, THA indications other than osteoarthritis, obesity, small head size, abnormal cup-placement angle, and prior spinal fusion have been associated with the risk of dislocation.^[7,12–19,27,30–32]^ The study period was defined as the acute period from the time of THA to the time of transfer from our hospital. Our results suggest that revision surgery and THA indications other than osteoarthritis indicate a high risk of dislocation during the early postoperative period. Fessy et al reported that low Devane Activity scores (low activity) indicate a higher risk of dislocation^[7]^; however, the relationship between preoperative activity levels and dislocation remains unclear. The dislocation group in our study also had significantly lower preoperative FIM motor, cognition, and locomotion status scores than the non-dislocation group. Previous studies have shown that dementia is associated with dislocation^[6]^ and that patients who have low preoperative activities of daily living, cognitive ability, and preoperative locomotion need special attention to prevent dislocation. The association between the approach and the occurrence of dislocation is controversial,^[32,33]^ and there is no consensus regarding the association between the use of cement and dislocation.^[34,35]^ However, this study showed significant differences related to the surgical approach and cement usage. Nevertheless, the influence of the surgical approach on dislocations is difficult to confirm because the approach differs depending on the patient’s condition, pathology, and surgeon.

More than half of the patients who experienced dislocations in this study underwent revision THA. Their hospital stay was approximately twice as long as that in than those without dislocations, and most patients were transferred to other hospitals, making dislocations a huge burden on patients. Therefore, more information is required to strengthen the measures to prevent dislocation. This study was a single-center retrospective cohort study with a sample size of only 6 patients who experienced dislocations. Therefore, large-scale, multicenter, joint research should be conducted in the future. However, this report provides useful information on measures to prevent dislocation during the acute hospital stay after THA.

## 5. Conclusions

Over a period of 5 years and 4 months, 6 of the 386 patients who underwent THA at our acute care hospital experienced postoperative dislocation. Revision THA, THA indications other than osteoarthritis, low FIM motor and cognitive scores on admission, and low preoperative locomotion status may contribute to the increased risk of dislocation in the early postoperative period. The study findings highlight the need to reassess in-hospital dislocation prevention strategies, particularly with regard to bed mobility and sleep positioning, and emphasize necessity of personalized measures in patients who have a complex medical history and functional disability.

## Acknowledgments

The authors thank the rehabilitation staff for their support with this study.

## Author contributions

**Conceptualization:** Fumihiko Suenaga, Tokio Kinoshita, Daisuke Nishiyama.

**Data curation:** Yoshinori Yasuoka, Kohei Minami, Daisuke Nishiyama.

**Formal analysis:** Yoshinori Yasuoka, Kohei Minami, Daisuke Nishiyama.

**Methodology:** Fumihiko Suenaga.

**Project administration:** Yukihide Nishimura, Hiroshi Yamada, Ken Kouda.

**Writing – original draft:** Fumihiko Suenaga, Tokio Kinoshita, Yoshinori Yasuoka, Kohei Minami.

**Writing – review & editing:** Tokio Kinoshita, Yukihide Nishimura, Daisuke Nishiyama, Hiroshi Yamada, Ken Kouda.
